# Prediabetes, Non-Dipping Profile and Hypertension—A Recipe for Increased Arterial Stiffness

**DOI:** 10.3390/biomedicines11041065

**Published:** 2023-04-01

**Authors:** Juraj Jug, Điđi Delalić, Valerija Bralić Lang, Tomislav Bulum, Ingrid Prkačin

**Affiliations:** 1 Health Center Zagreb-West, 10000 Zagreb, Croatia; 2 School of Medicine, University of Zagreb, 10000 Zagreb, Croatia; 3 Family medicine practice Valerija Bralić Lang, 10000 Zagreb, Croatia; 4 Vuk Vrhovac University Clinic for Diabetes, Endocrinology and Metabolic Diseases, Merkur University Hospital, 10000 Zagreb, Croatia; 5 Department of Internal E.R., Merkur University Hospital, 10000 Zagreb, Croatia

**Keywords:** arterial stiffness, blood pressure, hypertension, prediabetes, pulse wave velocity, target organ damage

## Abstract

Background: Pulse wave velocity (PWV) is a known predictor of target organ damage, cardiovascular disease and overall mortality. The aim of this study was to compare the PWV values in subjects with prediabetes, a non-dipper profile and arterial hypertension with their values in healthy subjects. Methods: A total of 301 subjects, aged 40–70 years, without diabetes mellitus were included in this cross-sectional study (150 with prediabetes). They underwent a 24 h ambulatory blood pressure monitoring (ABPM). Subjects were divided into three hypertension groups (A = healthy, B = controlled hypertension, C = uncontrolled hypertension). Dipping status was determined according to ABPM results, and PWV was measured by an oscillometric device. Prediabetes was defined as having 2 separate fasting plasma glucose (FPG) measurements between 5.6 and 6.9 mmol/L. Results: The highest PWV values were found in group C (9.60 ± 1.34 vs. 8.46 ± 1.01 in group B vs. 7.79 ± 1.10 in group A; *p* < 0.001), in subjects with prediabetes (8.98 ± 1.31 m/s vs. 8.26 ± 1.22 m/s; *p* < 0.001) and in prediabetic non-dippers among age groups (*p* = 0.05). In the multivariate regression model age, blood pressure, nocturnal indices and FPG were shown as independent predictors of PWV values. Conclusion: Significantly higher PWV values were found in subjects with prediabetes and non-dipping profiles in all three examined hypertension groups.

## 1. Introduction

Prediabetes (PreD) is a heterogeneous condition of impaired glucose metabolism in which the values do not meet the criteria for type 2 diabetes mellitus (T2DM) but are higher t the values considered normal. To have PreD, it is necessary to meet one or more set of criteria in at least two separate laboratory measurements (American Diabetes Association, ADA, 2017): fasting plasma glucose (FPG) 5.6–6.9 mmol/L, or HbA1c 5.7–6.4%, or blood glucose values by oral glucose load test 75 g glucose (OGTT) after 2 h 7.8–11.0 mmol/L [1]. Similarly, the guidelines of the European Society of Cardiology (ESC), in collaboration with the European Association for the Study of Diabetes (EASD), differ only in concentrations of FPG in which those of 6.1–6.9 mmol/L represent PreD. They both agree that testing is required in all persons over 45 years of age, and at any age if there is a high risk of developing prediabetes (BMI > 25 kg/m^2^, first-degree relative with diabetes, women who were diagnosed with gestational diabetes mellitus, hypertension, polycystic ovary syndrome, etc.) [1,2]. 

PreD is associated with a 20% higher risk of cardiovascular disease (CVD), especially carotid atherosclerosis and coronary artery calcification, while the association declines with aging, probably as a result of the influence of other risk factors [3,4,5]. Although some studies suggested that dietary or exercise interventions can prevent CVD in people with PreD, the risk should still be minimized by removing other risk factors for the development of CVD that are almost identical to those for the development of PreD [3]. 

Micro- and macrovascular damage are associated with PreD and are presented by greater arterial stiffness and faster progression of atherosclerosis. The gold standard for determining arterial stiffness is pulse wave velocity (PWV) using tonometry or oscillometric devices; arterial stiffness is a known predictor of target organ damage (TOD), CVD and overall mortality [6,7,8]. An increase in PWV of 1 m/s compared to the reference value for age and sex is associated with a 14% higher cardiovascular (CV) risk [9,10]. Notably, PWV values are physiologically higher in women in all age groups [11]. A study from Loehr et al. [12] on cohort members of the Atherosclerosis Risk in Communities (ARIC) study (4682 participants), demonstrated a significant association of PreD with brachial PWV. It is also known that hyperglycemia with hyperinsulinemia acutely increases PWV, heart rate and diastolic blood pressure (DBP) in healthy people, possibly due to an increase in sympathetic tone [13]. Reduction of PWV can be achieved by good control of arterial hypertension (AH) and other risk factors, thus reducing CV and mortality [14]. 

The absence of a drop in BP at night (non-dipping profile) is also a significant risk factor in the development of CVD, and it is known that an increase in systolic blood pressure (SBP) of 10 mmHg at night increases the risk of death by 21% [15]. Non-dipping profiles were observed more often in PreD only if chronic kidney disease was also present [16]. 

Hypertensive crisis is defined as a SBP above 180 mmHg and/or diastolic blood pressure (DBP) above 120 mmHg, which includes uncontrolled AH (HU, without TOD—eyes, brain, heart, aorta, kidneys) and hypertensive emergency (with acute TOD) [17,18,19]. In addition to BP control, rigorous control of all risk factors and screening of damage within the CV system is required. Although total CV risk reduction is the final goal of AH management, treatment according to guidelines, awareness of the seriousness of this problem and the need for a continuous individual and multidisciplinary approach are insufficient and call for change [20]. 

PreD and non-dipping profiles are significantly underestimated in clinical practice, leaving many patients with high cardiovascular risk and already existing TOD without adequate and timely therapy, especially if they have normal BP values [21,22]. PWV measurement is still relatively inaccessible, and according to current guidelines, its use in daily clinical practice is not recommended [17]. On the other hand, all other mentioned tests are recommended and available in a family medicine setting. 

### Aim of the Study

The aim of this study is to compare the PWV values in subjects with PreD, non-dipper profiles and arterial hypertension with their values in healthy subjects. We hypothesize that higher FPG values, higher BP levels and a non-dipper profile are associated with higher PWV values and higher CV risk, i.e., TOD. 

## 2. Methods

This cross-sectional study was conducted in four family medicine practices (FMP) of the Health Center Zagreb-West (groups A and B) and at the Emergency department of the Clinical Hospital Merkur (group C) in Zagreb, Croatia from January 2019 to December 2022. We completed an a priori power analysis and the power analysis resulted in 251; however, we recruited 301 Caucasian subjects (153 women and 148 men). Information about a patient’s medical history and used medication was collected from the electronic medical record (Medicus.Net system for FMP and Hospital Information System (BIS) in Clinical Hospital Merkur). Subjects were divided into three groups using the 24 h ambulatory blood pressure monitoring (ABPM) findings.

### 2.1. Groups Depending on Hypertensive Status

- Group A (healthy persons)—subjects were selected by simple random sampling from the list of insured persons in four FMPs without a diagnosis of AH (ICD-10: I10-I15) and with average BP values lower than 130/80 mmHg measured by ABPM.
- Group B (patients with AH without HU)—subjects from four FMPs with a diagnosis of AH (ICD-10: I10-I15) confirmed by ABPM without measured BP values higher than 180 mmHg and/or 120 mmHg in more than five individual measurements and data about HU in the medical history. AH is defined by exceeding the average of all measured BP values above 130 and/or 80 mmHg by APBM and/or taking at least one antihypertensive drug [16,22].
- Group C (patients with HU)—was formed by simple random sampling of patients from the database of patients from Clinical Hospital Merkur who sought emergency medical help due to high BP values. BP values upon arrival at the institution were measured after five minutes of quiet sitting with a one-minute interval between each measurement (three times), and ABPM was set upon discharge. Only those patients whose BP values were measured by a correctly performed ABPM of more than 180 and/or 120 mmHg in more than five individual measurements for 24 h were included.

### 2.2. Exclusion Criteria 

Exclusion criteria included hypertensive emergency, CV event in personal medical history, incomplete data, age below 40 and above 70 years, BMI > 50 kg/m^2^, atrial fibrillation, diabetes mellitus, chronic kidney disease G3b, G4 and G5, pregnancy, immunosuppressive, corticosteroid and/or biological therapy, chemotherapy regime in the last five years and expected life expectancy less than six months.

### 2.3. PWV and ABPM Measurements

A session of 24 h ABPM was performed on a special schedule (from 1 pm to 1 pm the next day, 80 BP measurements) using the BTL Cardiopoint^®^ (BTL, Marlborough, MA, USA) device using an adequate cuff placed on the lower half of the subject’s upper arm determined by the circumference of the middle part of the distal half of the upper arm (cuff S = 12 × 22 cm^2^ for circumference 22–26 cm, M = 16 × 30 cm^2^ for 27–34 cm, L = 16 × 36 cm^2^ for 35–44 cm, XL = 16 × 42 cm^2^ for 45–52 cm). All subjects were divided into three groups according to the percentage change in night BP value (dipper ≥10%, non-dipper 0–10% and inverse dipper <0%) [23,24]. In case of borderline BP values (e.g., more than five measured SBP values above 170 mmHg, etc.), duration of ABPM shorter than 20 h or less than 70% of correct measurements, ABPM was repeated two weeks after the first measurement [25].

PWV was measured with an Agedio^®^ B900 (IEM, Stolberg, Germany) oscillometric device at a room temperature of 20–25 °C with a suitable cuff in the same previously described manner as in the performance of ABPM. A total of 2 measurements were taken according to specific schedules with subjects (working days between 2 and 6 pm), between which there was a break of 30 s. At the first measurement, brachial SBP and DBP were determined, and at the second, PWV, central SBP (cSBP), central DBP (cDBP), pulse pressure (PP) and AIx. In case of imprecise measurement, the device reports an error, and the procedure was repeated within five minutes of the first measurement.

### 2.4. Other Procedures

Participants were recruited during their regular provider visits and then scheduled for ABPM, PWV measurements (within ten working days) and a blood draw. A blood draw was performed during the visit (emergency laboratory parameters for group C) or/and the morning after at 7 to 8.30 am (for groups A and B and other parameters for group C). Biochemical parameters in the examinee’s serum were determined by sampling 2 tubes of 8–10 mL of venous blood in the emergency hospital laboratory: the first was to determine hematocrit value, the concentration of creatinine, sodium ions, potassium ions and glucose, while the second was for triglycerides, HDL, LDL, total cholesterol and urate (since they could not be performed in an emergency laboratory). For other subjects, the same laboratory parameters were determined through the primary health care laboratory (including lipid status and urate concentration), and the fasting glucose value was repeated after two weeks. 

Acute organ damage in the case of HU was ruled out by standard biochemical and radiological examinations of the clinic (X-ray, CT, ultrasound with or without color doppler) and ECG. It includes acute kidney damage, ischemic or hemorrhagic stroke, acute coronary syndrome, acute pulmonary edema, acute left heart failure, hypertensive encephalopathy and aortic dissection. The glomerular filtration rate was assessed using CKD-EPI (Chronic Kidney Disease Epidemiology Collaboration) formula [26]. 

On the first visit, chronic antihypertensive and hypolipemic therapy was also recorded. Due to the possible effect on the measurements, the medications were not changed until all procedures were completed (if needed). Additionally, all participants were weighed in the office (anthropometric scale Tanita^®^ (TANITA, Stuttgart, Germany)) and had their height and waist circumference measured with a tailor’s tape measure. Prediabetes was defined according to the 2017 ADA criteria [1]. Metabolic syndrome was defined by the National Cholesterol Education Program (NCEP) Adult Treatment Panel III (ATP III) criteria updated by the American Heart Association and the National Heart Lung and Blood Institute in 2005 [27].

This study was approved by the Ethics Committee of the Health Center Zagreb-West (Number 251-12-02-21-19) and the Ethics Committee of the Merkur Clinical Hospital (Number 03/1-6063). All participants signed an informed consent to participate in the research.

### 2.5. Statistical Analysis

The sample size was calculated according to the expected arithmetic means of the PWV values and their standard deviations. Power analysis was performed a priori for ANOVA for special effects and interactions. With significance level α = 0.05, effect size (f) = 0.25, and a total of 251 respondents with equally large groups, ANOVA has a test power of β = 95% to recognize differences in two dependent variables between the three observed groups with one predictor [28]. Finally, 301 subjects were recruited. 

The normality of the data distribution was checked with the Kolmogorov-Smirnov test. Continuous variables (depending on the distribution) were analyzed with Student’s *t*-test, or Mann-Whitney U test, and categorical variables with Pearson’s χ2 test. ANOVA was used in the analysis of dependent variables. In post hoc analysis, we performed Tukey’s HSD test to check the differences between the groups. Variables that showed a statistically significant Pearson correlation with PWV were included in the linear regression model. The final linear regression model included PWV as a dependent variable and age, SBP, DBP, heart rate, nocturnal indices, FPG, and GFR as independent variables. A value of *p* < 0.05 is considered statistically significant. The program used for statistical analysis is Statistica v.12.0.

## 3. Results

In the studied groups, women were significantly older than men (58.11 vs. 54.59 years, *p* < 0.001), had higher PWV (8.74 vs. 8.44 m/s, *p* < 0.05) and AIx values (27.96 vs. 19.35%, *p* < 0.001), but lower SBP (130.85 vs. 136.97 mmHg, *p* < 0.01), DBP (77.26 vs. 84.46 mmHg, *p* < 0.001), triglycerides (1.40 vs. 1.85 mmol/L, *p* < 0.01), uric acid (294.50 vs. 378.52 umol/L, *p* < 0.001) and BMI values (27.24 vs. 28.45 kg/m^2^). Differences in FPG (5.68 vs. 5.85 mmol/L in men, *p* = 0.268) and other variables between sexes were not found. 

The division of subjects according to the results of ABPM resulted in 77 subjects in group A, 138 in group B and 86 in group C. Group C had a significantly higher number of patients with PreD (74.41%, *p* < 0.001) compared to the other groups (42.75 vs 35.06%, *p* = 0.269) and a significantly higher number of non-dippers (53.48%, *p* < 0.001) compared to other groups (32.61 vs. 29.87%, *p* = 0.653). Other differences are shown in column A in Table 1. There were 21 (15.22%) subjects newly diagnosed with HA in group B, and 23 (26.75%) in group C. A total of 8 (9.30%) subjects in group C were treated with only one antihypertensive drug. 

The number of dippers, specifically using nocturnal indices, were comparable between the healthy subjects and those with PreD (Table 1B). In the linear regression model, age, SBP, DBP, nocturnal indices and FPG proved to be independent predictors of PWV values (Table 2).

**Figure 1:**
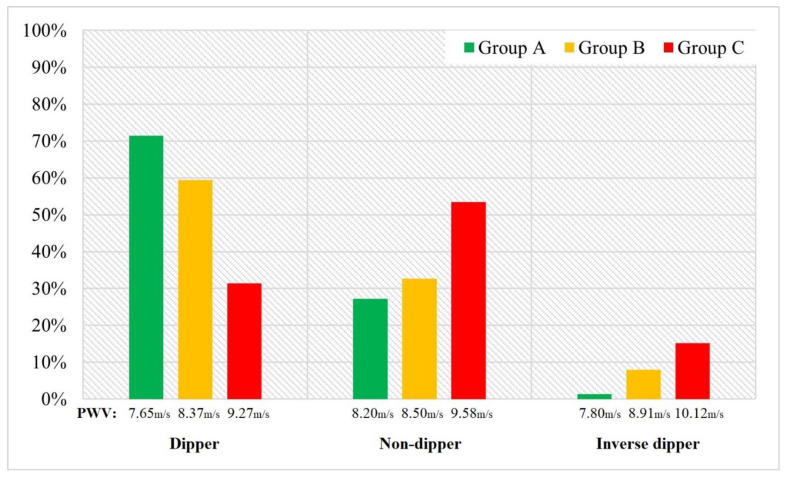
Ratio of subjects with certain dipping profile in examined groups and group pulse wave velocity (PWV) values. Group A = healthy subjects (N = 77); B = subjects with controlled hypertension (N = 138); C = subjects with uncontrolled hypertension (N = 86).

All dippers had the lowest average PWV values (8.23 m/s), followed by non-dippers with higher average PWV values (8.87 m/s, *p* < 0.001). The highest PWV values were measured in all inverse dippers (9.49 m/s, *p* < 0.001), especially in group C (10.12 m/s). All differences between the examined dipping profiles and hypertension groups are shown in Figure 1. 

**Figure 2:**
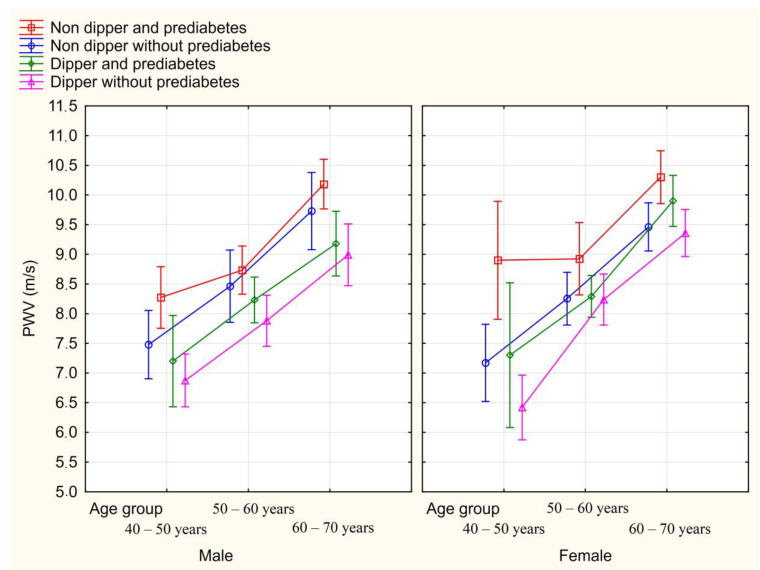
Pulse wave velocity (PWV) values among three age groups and genders depending on dipping profile and the presence of prediabetes (F = 0.86, *p* = 0.527 for all; F = 21.38, *p* < 0.001 for dipper and prediabetes groups).

PWV values were statistically much higher in subjects with both PreD and a non-dipper profile compared to subjects without PreD and with normal dipper profile in all three age groups (40–50 years: 8.41 vs. 6.69 m/s, *p* < 0.001; 50–60 years: 8.79 vs. 8.05 m/s, *p* < 0.01; 60–70 years: 10.24 vs. 9.22 m/s, *p* < 0.001). Although statistically insignificant, PWV values were slightly higher in non-dippers without PreD than in dippers with PreD (Figure 2), with no significant gender differences in post hoc analysis and the univariate test of significance (F = 0.80, *p* = 0.370). In PWV analysis among hypertension groups, subjects with PreD and a non-dipper profile had the highest values of all other group subjects without noticeable differences between genders (Table 3).

## 4. Discussion

In this study, PWV was significantly associated with the existence of PreD, nocturnal indices and BP values. PWV values were notably higher in PreD patients, especially in combination with a non-dipper profile in all three age and hypertensive groups. Special attention should be given to the group of apparently healthy subjects (group A) in which as many as 35% of them had PreD and 28% were non-dippers since their PWV values were even higher than in patients with well-controlled AH (group B) in our study. These subjects are often neglected in clinical practice because they seem to have a negligible CV risk, according to SCORE2 charts [29].

If we consider that higher PWV equals higher CV risk (TOD), then patients with PreD and non-dipping profiles in all hypertensive groups had the highest CV risk than all other subjects, which increases with aging [9,30,31]. Knowing that 9 to 50% of persons with PreD will develop T2DM in five years and 70% in ten years, the CV risk will additionally increase, because of which we have to start treating PreD and all CV risk factors immediately [32,33]. Therefore, Yasuno et al. [34] demonstrated high PP and PWV values as predictors of new-onset T2DM in hypertensive patients, independent of the effects of antihypertensive therapy and other risk factors. An American study from 2012 conducted by Mainous et al. [35] and using data from the National Ambulatory Medical Care Survey showed that only 23% of patients diagnosed with PreD were advised to change their lifestyle or were offered metformin. Many years before, the successfulness of lifestyle changes was shown by Tuomilehto et al. [36] who calculated that 22 subjects with PreD must be treated using non-pharmacological interventions in the primary care setting for one year (or five subjects for five years) to prevent one case of diabetes. Furthermore, obesity prevalence in children is growing worldwide and represents another crucial medical problem associated with increased arterial stiffness and BP values leading to accelerated atherosclerosis in early life [8]. Regardless of the age and CV risk, Zheng et al. [14] showed (on 20,355 participants) that avoiding smoking, alcohol non-consumption, low salt intake and increased physical activity (ideal CV health) can reduce PWV and the risk of cardiac and cerebral events.

Besides increased FPG, glycemic variability (GV) is associated with accelerated atherosclerosis even in non-diabetics, independently of conventional risk factors [37]. A study by Tateishi et al. [38] showed a greater GV impact on coronary endothelial dysfunction but no association with peripheral endothelial function. On the other hand, Yu et al. [39] observed CVD development and all-cause death over eight years in non-diabetic patients from a nationwide database of Koreans. They found that the risk of all-cause mortality and early death of CVD was significantly higher in the highest quartile of GV (24% and 21%, respectively). Although Foreman et al. [40] demonstrated the association between higher GV and greater PWV in 853 participants with T2DM, due to the very small number of studies which studied the relationship between GV and PWV, this association requires further investigation. Although our study did not investigate GV, high FPG was associated with significantly higher PWV. GV promotes inflammation and oxidative stress which causes the formation of cross-links of elastin and collagen [41,42]. Finally, the variability of many other CV risk factors (SBP, FPG, total cholesterol and BMI) was found to be an independent predictor of mortality and CV events [43]. Based on all the evidence, addressing all those metabolic risk factors is needed along with lifestyle changes in subjects with high PWV values (high CV risk).

A non-dipping profile is associated with impaired modulation of vascular smooth muscle tone during the night, increased PWV and CV risk [22,44]. The most common comorbidity connected with a non-dipping or inverse dipping profile is obstructive sleep apnea syndrome which should be quickly evaluated and treated [6]. Our results confirm the hypothesis that non-dipping status combined with PreD would lead to even greater PWV values, especially in subjects with uncontrolled arterial hypertension (group C), increasing their CV risk to high or very high [45]. We assume that PreD and non-dipper status did not affect PWV values in group B as expected (as in groups A and C) because of antihypertensive medication’s protective effect on the CV system (e.g., ACE inhibitors, ARBs) and good BP regulation [46].

A great problem of everyday clinical practice is the underutilization of CVD prevention. ESH-ESC Guidelines recommend using the SCORE2 model as a minimum requirement. However, TOD is not included in it, making the CV risk stratification imprecise [47]. For example, even 26% of subjects at the time of HU (group C) did not have a diagnosis of AH, while only 7% of subjects in group C were taking statin therapy despite the high CV risk and the guidelines. In our study, patients with uncontrolled AH had the highest PWV values in all age groups, independently of PreD and non-dipper profile, which is why we need to work on better care and treatment of such patients. More precise determination of total CV risk using oscillometric devices for PWV measurement in FMP should be the basis for timely recognition, rational diagnosis and better treatment of AH, reducing GV and FPG, and correcting dipping status and other CV risk factors to reduce the number of CV complications and improve the patient’s quality of life.

### Strengths and Limitations

The strength of this study is that PWV was also measured in healthy subjects included by strict criteria, always at a similar time of day (2–6 pm) with little differences in air temperature (20–25 °C), which can be extremely important for the accuracy of the measurement [48].

This study has several limitations. For defining PreD, we used average FPG from two measurements. HbA1c and values of albuminuria (an important parameter of TOD) could not be obtained due to practical reasons. In a small number of patients, the ABPM and PWV measurements were not performed on the same day due to the oscillometric device’s unavailability. The number of patients with an inverse dipping profile in different groups (especially in the age group 40–50 years) was too small (some were equal to zero) for good analysis using ANOVA. Accordingly, inverse dippers were not included in the ANOVA analysis.

## 5. Conclusions

Our findings showed significantly higher PWV values in subjects with PreD and non-dipping profile in all three examined hypertension groups, meaning these subjects already have higher CV risk and a greater probability of TOD. This study highlights the relevance of BP evaluation with ABPM in all subjects with PreD and the timely introduction of lifestyle changes and medication when needed. Future studies should assess the impact of lifestyle and medication interventions on PWV values in otherwise healthy subjects with PreD.

## Figures and Tables

**Table 1:** —Differences between healthy subjects (group A), patients with regulated arterial hypertension (group B) and patients with untreated hypertension (group C). B—Differences between healthy and prediabetic subjects (right side). All data are listed as number (percentage), mean ± standard deviation or median (interquartile range).

		A				B		
Variable	All Groups (N = 301)	Group A (N = 77)	Group B (N = 138)	Group C (N = 86)	*p*	Healthy (N = 151)	Prediabetes (N = 150)	*p*
Women	153 (50.83%)	49 (63.64%)	67 (48.55%)	37 (43.02%)	0.064 *	85 (56.29%)	68 (45.33%)	0.057 ^†^
Dippers	164 (54.48%)	55 (71.43%)	82 (59.42%)	27 (31.40%)	<0.001 *	87 (57.61%)	77 (51.33%)	0.274 ^†^
Metabolic syndrome	90 (29.90%)	3 (3.89%)	39 (28.26%)	48 (55.81%)	<0.001 *	20 (13.24%)	69 (46.00%)	<0.001 ^†^
Antihypertensive therapy	180 (80.36%) ^ϴ^	/	117 (84.78%)	63 (73.25%)	0.119 *	85 (56.29%)	98 (65.33%)	0.108 ^†^
Statin therapy	28 (9.31%)	3 (3.89%)	19 (13.77%)	6 (6.98%)	<0.05 *	13 (8.61%)	17 (11.33%)	0.430 ^†^
Current smoking	110 (36.54%)	28 (36.37%)	45 (32.61%)	37 (43.02%)	<0.05 *	46 (30.46%)	64 (42.66%)	<0.05 ^†^
Age (years)	56.37 ± 7.94	54.68 ± 7.65	57.16 ± 7.61	56.72 ± 8.61	0.069 *	55.59 ± 8.56	57.53 ± 7.01	<0.05 ^‡^
Average SBP (mmHg)	133.89 ±16.72	117.58 ± 7.61	134.02 ± 10.52	149.41 ± 16.52	<0.001 *	130.90 ± 15.62	137.51 ± 17.34	<0.001 ^‡^
Average DBP (mmHg)	80.82 ± 11.95	71.71 ± 6.73	79.48 ± 8.35	91.80 ± 12.39	<0.001 *	79.36 ± 11.80	82.52 ± 12.07	<0.05 ^‡^
Average MAP (mmHg)	98.51 ± 12.81	87.00 ± 6.53	97.66 ± 8.37	111.01 ± 12.26	<0.001 *	96.54 ± 12.46	100.85 ± 12.97	<0.01 ^‡^
Nocturnal indices (%)	10.83 (4.47–15.44)	12.12 (7.08–17.47)	11.16 (5.26–16.38)	6.18 (2.18–12.14)	<0.001 ^§^	10.66 (4.76–15.83)	10.01 (4.17–14.81)	0.358 ^§^
cSBP (mmHg)	129.82 ± 20.87	115.02 ± 11.09	124.34 ± 13.10	153.39 ± 19.06	<0.001 *	124.61 ± 18.29	135.60 ± 22.24	<0.001 ^‡^
cDBP (mmHg)	90.04 ± 15.54	79.25 ± 9.84	87.50 ± 11.27	104.65 ± 15.41	<0.001 *	87.27 ± 14.62	93.07 ± 16.13	<0.01 ^‡^
PP (mmHg)	39.94 ± 12.57	36.30 ± 11.68	36.85 ± 8.81	48.81 ± 14.29	<0.001 *	37.24 ± 11.09	42.98 ± 13.42	<0.001 ^‡^
Heart rate (/min)	73.43 ± 9.24	71.77 ± 7.83	72.41 ± 9.55	76.69 ± 9.28	<0.001 *	73.30 ± 9.91	73.71 ± 8.68	0.444 ^‡^
GFR (ml/min/1.73 m^2^)	89.83 ± 14.52	90.70 ± 13.51	89.59 ± 13.43	89.43 ± 17.01	0.828 *	90.46 ± 13.44	89.19 ± 15.56	0.449 ^‡^
FPG (mmol/L)	5.76 ± 1.33	5.39 ± 0.61	5.56 ± 0.77	6.41 ± 1.09	<0.001 *	5.04 ± 0.32	6.48 ± 0.56	<0.001 ^‡^
Potassium (mmol/L)	4.27 ± 0.41	4.37 ± 0.34	4.30 ± 1.18	4.12 ± 0.45	<0.001 *	4.30 ± 0.41	4.23 ± 0.40	0.118 ^‡^
Total cholesterol (mmol/L)	5.93 ± 1.18	5.94 ± 1.11	5.77 ± 1.18	6.16 ± 1.23	0.062 *	5.78 ± 1.20	6.07 ± 1.15	<0.05 ^‡^
Non-HDL c (mmol/L)	4.44 ± 1.39	4.23 ± 1.09	4.34 ± 1.13	4.77 ± 1.17	<0.05 *	4.29 ± 1.16	4.58 ± 1.57	<0.01 ^‡^
Triglycerides (mmol/L)	1.62 ± 0.97	1.1 (0.9–1.6)	1.4 (1.1–1.8)	1.5 (1.1–2.2)	<0.05 ^§^	1.3 (0.9–1.7)	1.5 (1.1–2.1)	<0.01 ^§^
Uric acid (umol/L)	335.99 ± 88.34	295.49 ± 70.16	338.36 ± 84.59	373.39 ± 96.86	<0.001 *	325.36 ± 83.07	348.24 ± 92.93	0.063 ^‡^
BMI (kg/m^2^)	28.15 ± 6.94	24.71 ± 3.42	28.18 ± 4.74	30.34 ± 4.66	<0.001 *	27.24 ± 5.12	28.45 ± 4.51	<0.05 ^‡^
WC (cm)	94.3 ± 3.3	88.4 ± 2.8	94.2 ± 3.1	101.2 ± 4.6	<0.001 *	94.0 ± 2.4	99.0 ± 3.4	<0.01 ^‡^
PWV (m/s)	8.59 ± 1.32	7.79 ± 1.10	8.46 ± 1.01	9.60 ± 1.34	<0.001 *	8.26 ± 1.22	8.98 ± 1.31	<0.001 ^‡^
AIx (%)	22 (15–31)	21 (12–34)	21 (13–31)	24 (21–30)	<0.05 ^§^	22 (13–31)	23 (18–32)	0.247 ^§^

SB*p* = systolic blood pressure; DB*p* = diastolic blood pressure; MA*p* = mean arterial pressure; GFR = glomerular filtration rate; FPG = fasting plasma glucose; PWV = pulse wave velocity; cSB*p* = central systolic blood pressure; cDB*p* = central diastolic blood pressure; P*p* = pulse pressure; AIx = augmentation index; WC = waist circumference; ^ϴ^ = without group A. * ANOVA; ^†^ Pearson χ^2^ test; ^‡^ Student *t*-test; ^§^ Mann–Whitney test.

**Table 2:** Evaluation of parameters affecting PWV by linear regression analysis. Regression summary for dependent variable PWV: R = 0.839; R² = 0.705; Adjusted R² = 0.698; F (7.291) = 99.507; *p* < 0.001; Standard Error of estimate: 0.721; N = 301.

	B	Standard Error	T	*p*
Intercept		0.694	−3.495	<0.001
Age	0.736	0.035	20.759	<0.001
SBP	0.222	0.049	4.539	<0.001
DBP	0.109	0.051	2.135	0.034
Nocturnal indices	−0.107	0.459	−3.280	0.001
Heart rate	0.040	0.034	1.189	0.235
GFR	−0.048	0.034	−1.416	0.158
FPG	0.143	0.032	4.433	<0.001

SB*p* = systolic blood pressure; DB*p* = diastolic blood pressure; GFR = glomerular filtration rate; FPG = fasting plasma glucose; PWV = pulse wave velocity.

**Table 3:** Hypertension groups and PWV values depending on dipping status, prediabetes (PreD) and gender. Values represent means ± standard deviation and were analyzed by one-way ANOVA divided by groups.

	Gender	Non-Dipper and PreD (N = 72)	Dipper and PreD (N = 64)	Non-Dipper without PreD (N = 77)	Dipper without PreD (N = 87)	*p*
Group A (Healthy subjects)	Male	8.17 ± 1.04	7.90 ± 0.56	7.82 ± 1.14	7.22 ± 0.99	0.904
	Female	9.07 ± 1.04	8.60 ± 0.93	8.34 ± 0.66	7.70 ± 1.30	
Group B (Subjects with controlled hypertension)	Male	8.37 ± 1.16	8.31 ± 1.38	8.22 ± 0.93	7.98 ± 0.92	0.308
	Female	8.90 ± 0.23	8.62 ± 0.90	8.82 ± 0.91	8.32 ± 1.07	
Group C (Subjects with uncontrolled hypertension)	Male	9.42 ± 1.25	9.05 ± 1.50	8.60 ± 0.66	8.12 ± 0.59	0.572
	Female	10.37 ± 1.42	9.90 ± 0.28	10.10 ± 0.79	9.75 ± 1.04	
All groups	Male	9.16 ± 1.42	8.46 ± 1.29	8.35 ± 0.91	7.81 ± 1.01	0.769
	Female	9.71 ± 1.39	8.60 ± 1.16	8.52 ± 1.17	8.31 ± 1.30	

## Data Availability

The data presented in this study are available on request from the corresponding author.

## References

[1] American Diabetes Association 2 (2017). Classification and Diagnosis of Diabetes. Diabetes Care.

[2] Ryden L., Grant P.J., Anker S.D., Berne C., Cosentino F., Danchin N., Deaton C., Escaned J., Hammes H.P., Authors/Task Force Members (2014). ESC Guidelines on Diabetes, Pre-Diabetes, and Cardiovascular Diseases Developed in Collaboration with the EASD. The Task Force on Diabetes, Pre-Diabetes, and Cardiovascular Diseases of the European Society of Cardiology (ESC) and Developed in Collaboration with the European Association for the Study of Diabetes (EASD). Rev. Esp. Cardiol. Engl. Ed..

[3] Færch K., Vistisen D., Johansen N.B., Jørgensen M.E. (2014). Cardiovascular Risk Stratification and Management in Pre-Diabetes. Curr. Diabetes Rep..

[4] Ford E.S., Zhao G., Li C. (2010). Pre-Diabetes and the Risk for Cardiovascular Disease. J. Am. Coll. Cardiol..

[5] Gupta A.K., Greenway F.L., Cornelissen G., Pan W., Halberg F. (2008). Prediabetes Is Associated with Abnormal Circadian Blood Pressure Variability. J. Hum. Hypertens..

[6] Roth G.A., Mensah G.A., Johnson C.O., Addolorato G., Ammirati E., Baddour L.M., Barengo N.C., Beaton A.Z., Benjamin E.J., Benziger C.P. (2020). Global Burden of Cardiovascular Diseases and Risk Factors, 1990–2019. J. Am. Coll. Cardiol..

[7] de Rezende Mikael  L., de Paiva A.M.G., Gomes M.M., Sousa A.L.L., Jardim P.C.B.V., de Oliveira Vitorino P.V., Euzébio M.B., de Sousa W.M., Barroso W.K.S. (2017). Vascular Aging and Arterial Stiffness. Arq. Bras. Cardiol..

[8] Mihuta M.S., Paul C., Borlea A., Cepeha C.M., Velea I.P., Mozos I., Stoian D. (2022). The Oscillometric Pulse Wave Analysis Is Useful in Evaluating the Arterial Stiffness of Obese Children with Relevant Cardiometabolic Risks. J. Clin. Med..

[9] Vlachopoulos C., Aznaouridis K., Stefanadis C. (2010). Prediction of Cardiovascular Events and All-Cause Mortality with Arterial Stiffness. J. Am. Coll. Cardiol..

[10] Vasan R.S., Short M.I., Niiranen T.J., Xanthakis V., DeCarli C., Cheng S., Seshadri S., Mitchell G.F. (2019). Interrelations between Arterial Stiffness, Target Organ Damage, and Cardiovascular Disease Outcomes. J. Am. Heart Assoc..

[11] Aristizábal-Ocampo D., Espíndola-Fernández D., Gallo-Villegas J. (2019). Pulse Wave Velocity Reference Values in 3,160 Adults Referred to a Hypertension Clinic for 24-Hour Ambulatory Blood Pressure Monitoring. Clin. Exp. Hypertens..

[12] Loehr L.R., Meyer M.L., Poon A.K., Selvin E., Palta P., Tanaka H., Pankow J.S., Wright J.D., Griswold M.E., Wagenknecht L.E. (2016). Prediabetes and Diabetes Are Associated with Arterial Stiffness in Older Adults: The ARIC Study. Am. J. Hypertens..

[13] Horton W.B., Jahn L.A., Hartline L.M., Aylor K.W., Patrie J.T., Barrett E.J. (2021). Insulin Increases Central Aortic Stiffness in Response to Hyperglycemia in Healthy Humans: A Randomized Four-Arm Study. Diabetes Vasc. Dis. Res..

[14] Zheng X., Zhang R., Liu X., Zhao H., Liu H., Gao J., Wu Y., Wu S. (2017). Association between Cumulative Exposure to Ideal Cardiovascular Health and Arterial Stiffness. Atherosclerosis.

[15] Dolan E., Stanton A.A.N., Thijs L.M.S. (2005). Superiority of Ambulatory over Clinic Blood Pressure Measurement in Predicting Mortality: The Dublin Outcome Study. Hypertension.

[16] Obayashi K., Saeki K., Kurumatani N. (2016). Nighttime BP in Elderly Individuals with Prediabetes/Diabetes with and without CKD: The HEIJO-KYO Study. Clin. J. Am. Soc. Nephrol..

[17] Mancia G., Rosei E.A., Azizi M., Burnier M., Clement D.L., Coca A., de Simone G., Dominiczak A., Kahan T., Mahfoud F. (2018). 2018 ESC/ESH Guidelines for the Management of Arterial Hypertension. Eur. Heart J..

[18] Patel K.K., Young L., Howell E.H., Hu B., Rutecki G., Thomas G., Rothberg M.B. (2016). Characteristics and Outcomes of Patients Presenting with Hypertensive Urgency in the Office Setting. JAMA Intern. Med..

[19] Astarita A., Covella M., Vallelonga F., Cesareo M., Totaro S., Ventre L., Aprà F., Veglio F., Milan A. (2020). Hypertensive Emergencies and Urgencies in Emergency Departments: A Systematic Review and Meta-Analysis. J. Hypertens..

[20] Levy P.D., Mahn J.J., Miller J., Shelby A., Brody A., Davidson R., Burla M.J., Marinica A., Carroll J., Purakal J. (2015). Blood Pressure Treatment and Outcomes in Hypertensive Patients without Acute Target Organ Damage: A Retrospective Cohort. Am. J. Emerg. Med..

[21] Zand A., Ibrahim K., Patham B. (2018). Prediabetes: Why Should We Care?. Methodist DeBakey Cardiovasc. J..

[22] Cicek Y., Durakoglugil M.E., Kocaman S.A., Cetin M., Erdogan T., Dogan S., Ugurlu Y., Canga A. (2013). Non-Dipping Pattern in Untreated Hypertensive Patients Is Related to Increased Pulse Wave Velocity Independent of Raised Nocturnal Blood Pressure. Blood Press..

[23] O’Brien E., Parati G., Stergiou G., Asmar R., Beilin L., Bilo G., Clement D., de la Sierra A., de Leeuw P., Dolan E. (2013). European Society of Hypertension Position Paper on Ambulatory Blood Pressure Monitoring. J. Hypertens..

[24] Fagard R.H. (2009). Dipping Pattern of Nocturnal Blood Pressure in Patients with Hypertension. Expert Rev. Cardiovasc. Ther..

[25] Gaborieau V., Delarche N., Gosse P. (2008). Ambulatory Blood Pressure Monitoring versus Self-Measurement of Blood Pressure at Home: Correlation with Target Organ Damage. J. Hypertens..

[26] Levey A.S., Stevens L.A., Schmid C.H., Iii A.F.C., Feldman H.I., Kusek J.W., Eggers P., Coresh J. (2009). A New Equation to Estimate Glomerular Filtration Rate. Ann. Intern. Med..

[27] Howard W.J. (2006). Diagnosis and Management of the Metabolic Syndrome: An American Heart Association/National Heart, Lung, and Blood Institute Scientific Statement. Yearb. Endocrinol..

[28] Cohen J. (1988). Statistical Power Analysis for the Behavioral Sciences.

[29] SCORE2 Working Group and ESC Cardiovascular Risk Collaboration (2021). SCORE2 Risk Prediction Algorithms: New Models to Estimate 10-Year Risk of Cardiovascular Disease in Europe. Eur. Heart J..

[30] Gagliardino J.J., Salazar M.R., Espeche W.G., Tolosa Chapasian P.E., Gomez Garizoain D., Olano R.D., Stavile R.N., Balbín E., Martinez C., Leiva Sisnieguez B.C. (2021). Arterial Stiffness: Its Relation with Prediabetes and Metabolic Syndrome and Possible Pathogenesis. J. Clin. Med..

[31] Kim J.M., Kim S.S., Kim I.J., Kim J.H., Kim B.H., Kim M.K., Lee S.H., Lee C.W., Kim M.C., Ahn J.H. (2020). Arterial Stiffness Is an Independent Predictor for Risk of Mortality in Patients with Type 2 Diabetes Mellitus: The REBOUND Study. Cardiovasc. Diabetol..

[32] Bertram M.Y., Vos T. (2010). Quantifying the Duration of Pre-Diabetes. Aust. N. Z. J. Public Health.

[33] Zhang X., Gregg E.W., Williamson D.F., Barker L.E., Thomas W., Bullard K.M., Imperatore G., Williams D.E., Albright A.L. (2010). A1C Level and Future Risk of Diabetes: A Systematic Review. Diabetes Care.

[34] Yasuno S., Ueshima K., Oba K., Fujimoto A., Hirata M., Ogihara T., Saruta T., Nakao K. (2010). Is Pulse Pressure a Predictor of New-Onset Diabetes in High-Risk Hypertensive Patients?. Diabetes Care.

[35] Mainous A.G., Tanner R.J., Baker R. (2016). Prediabetes Diagnosis and Treatment in Primary Care. J. Am. Board Fam. Med..

[36] Tuomilehto J., Lindström J., Eriksson J.G., Valle T.T., Hämäläinen H., Ilanne-Parikka P., Keinänen-Kiukaanniemi S., Laakso M., Louheranta A., Rastas M. (2001). Prevention of Type 2 Diabetes Mellitus by Changes in Lifestyle among Subjects with Impaired Glucose Tolerance. N. Engl. J. Med..

[37] Nusca A., Tuccinardi D., Albano M., Cavallaro C., Ricottini E., Manfrini S., Pozzilli P., Di Sciascio G. (2018). Glycemic Variability in the Development of Cardiovascular Complications in Diabetes. Diabetes Metab. Res. Rev..

[38] Tateishi K., Saito Y., Kitahara H., Kobayashi Y. (2022). Impact of Glycemic Variability on Coronary and Peripheral Endothelial Dysfunction in Patients with Coronary Artery Disease. J. Cardiol..

[39] Yu J.H., Han K., Park S., Lee D.Y., Nam G.E., Seo J.A., Kim S.G., Baik S.H., Park Y.G., Kim S.M. (2019). Effects of Long-Term Glycemic Variability on Incident Cardiovascular Disease and Mortality in Subjects without Diabetes: A Nationwide Population-Based Study. Medicine.

[40] Foreman Y.D., van Doorn W.P.T.M., Schaper N.C., van Greevenbroek M.M.J., van der Kallen C.J.H., Henry R.M.A., Koster A., Eussen S.J.P.M., Wesselius A., Reesink K.D. (2021). Greater Daily Glucose Variability and Lower Time in Range Assessed with Continuous Glucose Monitoring Are Associated with Greater Aortic Stiffness: The Maastricht Study. Diabetologia.

[41] Ceriello A., Kilpatrick E.S. (2013). Glycemic Variability: Both Sides of the Story. Diabetes Care.

[42] Prenner S.B., Chirinos J.A. (2015). Arterial Stiffness in Diabetes Mellitus. Atherosclerosis.

[43] Kim M.K., Han K., Park Y.-M., Kwon H.-S., Kang G., Yoon K.-H., Lee S.-H. (2018). Associations of Variability in Blood Pressure, Glucose and Cholesterol Concentrations, and Body Mass Index with Mortality and Cardiovascular Outcomes in the General Population. Circulation.

[44] Jerrard-Dunne P., Mahmud A., Feely J. (2007). Circadian Blood Pressure Variation: Relationship between Dipper Status and Measures of Arterial Stiffness. J. Hypertens..

[45] Ben-Shlomo Y., Spears M., Boustred C., May M., Anderson S.G., Benjamin E.J., Boutouyrie P., Cameron J., Chen C.-H., Cruickshank J.K. (2014). Aortic Pulse Wave Velocity Improves Cardiovascular Event Prediction. J. Am. Coll. Cardiol..

[46] Li X., Chang P., Wang Q., Hu H., Bai F., Li N., Yu J. (2020). Effects of Angiotensin-Converting Enzyme Inhibitors on Arterial Stiffness: A Systematic Review and Meta-Analysis of Randomized Controlled Trials. Cardiovasc. Ther..

[47] Redon J. (2016). Global Cardiovascular Risk Assessment: Strengths and Limitations. High Blood Press. Cardiovasc. Prev..

[48] Podrug M., Šunjić B., Bekavac A., Koren P., Đogaš V., Mudnić I., Boban M., Jerončić A. (2023). The Effects of Experimental, Meteorological, and Physiological Factors on Short-Term Repeated Pulse Wave Velocity Measurements, and Measurement Difficulties: A Randomized Crossover Study with Two Devices. Front. Cardiovasc. Med..

